# Role of BET Proteins in Inflammation and CNS Diseases

**DOI:** 10.3389/fmolb.2021.748449

**Published:** 2021-09-16

**Authors:** Lei Liu, Changjun Yang, Eduardo Candelario-Jalil

**Affiliations:** Department of Neuroscience, McKnight Brain Institute, University of Florida, Gainesville, FL, United States

**Keywords:** neuroinflammation, proteolysis-targeting chimera, neurological diseases, stroke, multiple sclerosis, spinal cord injury, seizure, Alzheimer’s disease

## Abstract

Bromodomain and extra-terminal domain (BET) proteins consist of four mammalian members (BRD2, BRD3, BRD4, and BRDT), which play a pivotal role in the transcriptional regulation of the inflammatory response. Dysregulated inflammation is a key pathological process in various CNS disorders through multiple mechanisms, including NF-κB and Nrf2 pathways, two well-known master regulators of inflammation. A better mechanistic understanding of the BET proteins’ role in regulating the inflammatory process is of great significance since it could reveal novel therapeutic targets to reduce neuroinflammation associated with many CNS diseases. In this minireview, we first outline the structural features of BET proteins and summarize genetic and pharmacological approaches for BET inhibition, including novel strategies using proteolysis-targeting chimeras (PROTACs). We emphasize *in vitro and in vivo* evidence of the interplay between BET proteins and NF-κB and Nrf2 signaling pathways. Finally, we summarize recent studies showing that BET proteins are essential regulators of inflammation and neuropathology in various CNS diseases.

## Introduction

The bromodomain and extra-terminal domain (BET) family is evolutionarily conserved and plays a pivotal role in the transcriptional regulation of inflammation (Belkina and Denis, 2012; Marmorstein and Zhou, 2014). BET proteins consist of the ubiquitously expressed bromodomain-containing protein (BRD) 2, 3, 4, and the testis-specific isoform BRDT. Over the past decade, BET proteins have attracted intense interest in academia and the pharmaceutical industry as new therapeutic targets for inflammation-associated CNS diseases and cancer. Since the discovery of BET proteins in the 1990s (Haynes et al., 1992), subsequent work with pharmacologic and genetic tools focused on BET proteins’ biological function and their relevance in diseases. In the 2000s, early studies showed that loss of either BRD2 or BRD4 is lethal in mice (Houzelstein et al., 2002; Shang et al., 2009). Since 2010, significant progress has been made in understanding the crucial role that BET proteins play as enhancers of inflammatory gene transcription. Emerging evidence has recently revealed that BET proteins coordinately regulate an expansive range of inflammatory genes through NF-kB or Nrf2 pathway. A complete understanding of BET protein’s role in regulating inflammation and its contribution to CNS pathologies is significant.

Against this background, the present minireview outlines the structural characteristics of BET proteins, genetic and pharmacological inhibition approaches in discovering BET biology, BET inflammatory regulation, and its role in different CNS diseases. In particular, we present evidence of the interplay between BET proteins and the transcription factors NF-kB and Nrf2, which are essential regulators of the inflammatory process.

## Structural Domains of BET Proteins

**FIGURE 1:**
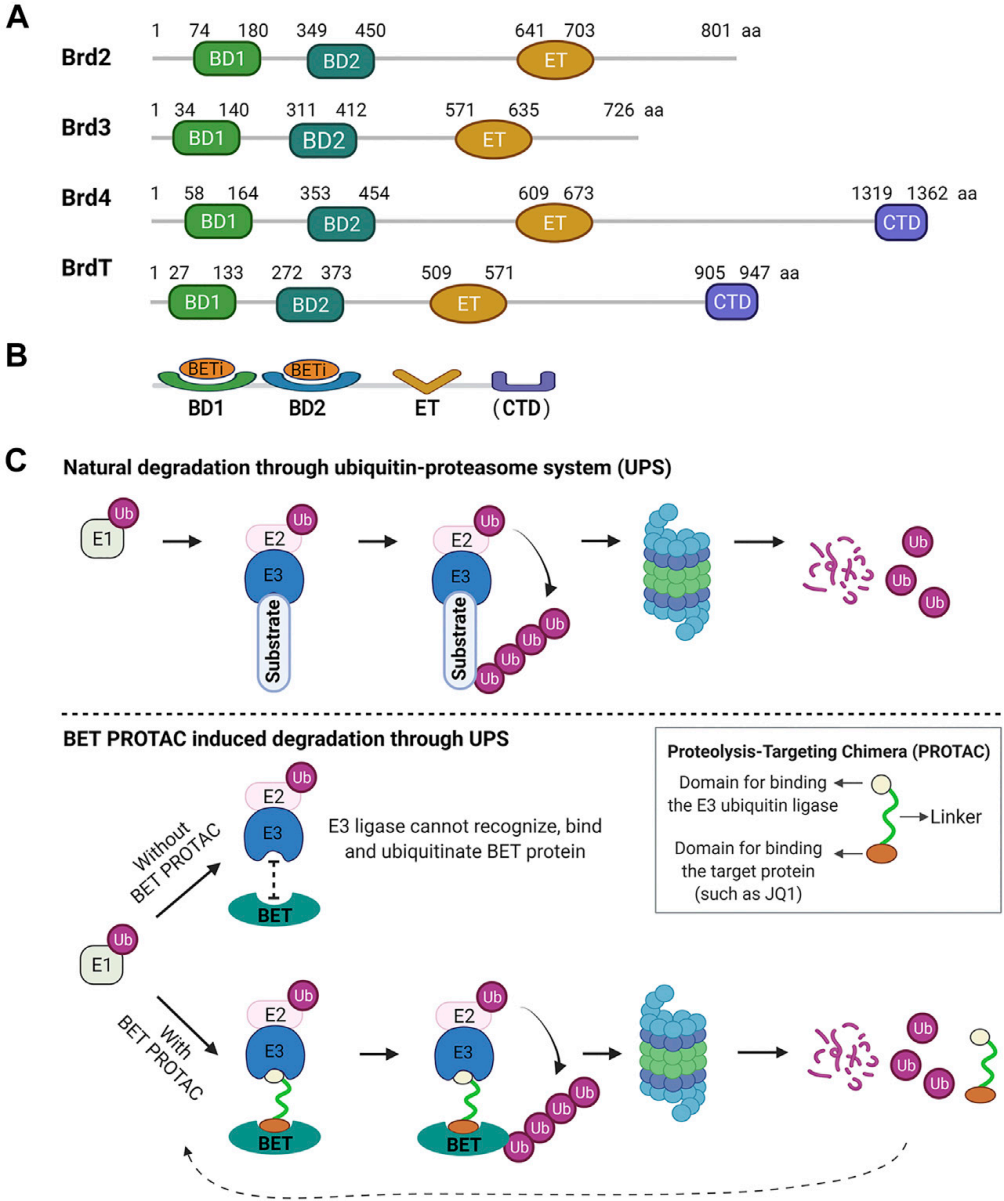
Human BET family structural domains and mechanisms of inhibition. **(A)** Structural domains of BET proteins. All BET proteins share two tandem N-terminal bromodomains (BD1 and BD2) and an extra-terminal (ET) domain. In addition, Brd4 and BrdT contain a unique C-terminal domain (CTD). The numbers refer to the amino acid boundaries of each domain for the human BET proteins. The alignment of amino acid (aa) sequences is from the public protein sequence database, GenPept, NCBI Reference Sequence: Brd2, NP_005095.1; Brd3, NP_031397.1; Brd4, NP_490,597.1; BrdT, NP_001229734.2. **(B)** To date, all reported BET inhibitors (BETis) target BDs on BET proteins. **(C)** BET PROTAC mechanism. The E1 enzyme activates ubiquitin (Ub) and initiates its transfer to a target substrate through the E1-E2-E3 cascade. Then, the target substrate tagged by ubiquitin is degraded by the proteasome. Without BET PROTAC, BET protein is not recognizable for the ubiquitination process. BET PROTAC molecules can bind the target BET protein and the E3 ubiquitin ligase together, and such binding ubiquitinates the target protein, making it available for subsequent proteasomal degradation.

BET proteins share a common domain architecture, characterized by the presence of two tandem N-terminal bromodomains (BD1 and BD2) and a unique extra-terminal (ET) domain (Shi and Vakoc, 2014) (Figure 1A). Other BD-containing proteins lack this double-barrel feature. In addition, BRD4 and BRDT contain a unique C-terminal domain (CTD). Bromodomains (BDs) are protein interaction modules of ∼110 amino acids that specifically recognize acetylated lysine in histones and other proteins with different functions and are essential for transcriptional regulation and chromatin remodeling (Dhalluin et al., 1999; Filippakopoulos et al., 2012). Since their initial discovery in 1992, 61 BDs have been identified in 46 different human proteins (Haynes et al., 1992; Cochran et al., 2019) and are clustered into eight families according to their sequence or structural similarities (Haynes et al., 1992; Filippakopoulos et al., 2012). As family II of BDs, the BET family is the most intensely studied.

BD1 and BD2 retain a high sequence identity (75%) or structural similarity across BET members (Ferri et al., 2016). Most BET inhibitors bind to both BD1 and BD2. Therefore, they show selectivity for all BET proteins in a concentration-dependent manner. In contrast, BD1 and BD2 within a given BET member have low sequence similarity (45%), indicating that they may have different roles in regulating the expression of BET-sensitive genes. Indeed, most recent selective BET inhibition studies have outlined the functional difference between BD1 and BD2 in biology and therapy; BD2 displays a more selective inflammatory phenotype in producing critical pro-inflammatory mediators (Jahagirdar et al., 2017; Gilan et al., 2020). ET domain is a protein interaction motif with a highly conserved region of ∼80 amino acids, exhibiting more than 80% identity among BRD2, 3, and 4 (Rahman et al., 2011). This domain carries out a regulatory function by recruiting specific effector proteins (Werner et al., 2020). CTM is uniquely present in BRD4 and BRDT functions in modulating positive transcription elongation factor activity (Yang et al., 2005).

## Pharmacological and Genetic Approaches Help to Illuminate BET Proteins’ Function

BET proteins are attractive targets from a chemical and structural perspective owing to their unique properties that mechanistically link bromodomain recognition with transcriptional regulation. This feature has prompted a wave of pharmacological and genetic approaches to understand BET proteins’ biological function(s) and their role in diseases.

**BET inhibitors-Focus on BD inhibition.** So far, all reported BET inhibitors (BETis) focus on BET BDs, a highly tractable small molecules target (Figure 1B). In 2005 and 2006, two important proof-of-concept studies demonstrated the feasibility of small-molecule inhibition of BDs. In 2010, the landmark studies of potent and selective BETis, JQ1 and I-BET, thoroughly characterized small-molecule inhibitors targeting BET BDs and revealed the crucial role of BET proteins in anti-inflammatory and anticancer activities (Filippakopoulos et al., 2010; Nicodeme et al., 2010). Like JQ1 and I-BET, the first-generation BETis bind with high affinity to both BDs of each BET protein, therefore competitively disrupting the binding of BDs with acetylated lysine residues. Although they are non-selective for any specific BET protein, these BETis exhibit a much higher affinity for BRD4 than for other BET family members. For instance, JQ1 was shown to be selective for BET BDs with higher affinity for BRD4 (50 nM for BD1) and lower affinity for other BET members (60–190 nM) (Filippakopoulos et al., 2010; Ferri et al., 2016). Therefore, BRD4 functional regulation is the primary effect of these BET inhibitors (Filippakopoulos et al., 2010; Kanno et al., 2014). Emerging strategies focus on discovering compounds that selectively or cooperatively target the tandem BET BD1 and BD2, which may elucidate the differential roles of BD1 and BD2 (Sheppard et al., 2020). A recent study suggests that BD1 and BD2 have separate and distinct roles in transcriptional regulation (Gilan et al., 2020). To date, more than 10 BETis, including the BD2 selective inhibitor ABBV-744, have progressed to human clinical trials at different phases (Cochran et al., 2019; Sheppard et al., 2020). The discovery of BD inhibitors and their biological and therapeutic potential have been summarized in recent excellent comprehensive reviews (Filippakopoulos and Knapp, 2014; Ferri et al., 2016; Cochran et al., 2019).

**BET PROTACs-Induction of BET protein degradation through the ubiquitin-proteasome system (UPS).** An appealing approach to block multi-domain protein function is to delete the protein completely (Cochran et al., 2019), which allows rapid progress in understanding cellular protein functions and their relevance in diseases. This approach can be accomplished by either genetic manipulation (such as knockout or knockdown) or targeting protein degradation with proteolysis-targeting chimeras (PROTACs). PROTACs represent a leading strategy for targeting protein degradation through the UPS, initially reported in 2001 (Sakamoto et al., 2001). PROTACs are heterobifunctional small molecules composed of two linked domains for binding the target protein and the E3 ubiquitin ligase. The binding of both moieties allows the target protein to be tagged with ubiquitin, making it available for subsequent degradation by the proteasomal machinery (Figure 1C). The chemical probes like pan-BETis structural class can recognize and recruit BET BDs and serve as ideal PROTAC anchors for BET proteins. In 2015, the first BET-targeted PROTACs dBET1, MZ1, and ARV-825 were described, containing a BRD4 BD binding moiety (i.e., JQ1 or OTX015) and an E3 ligase binding moiety. These BET PROTACs recognize and recruit the E3 ubiquitin ligase to targeted BRD4, leading to rapid and efficient deletion of BET proteins, especially BRD4 *in vitro* and *in vivo* (Lu et al., 2015; Winter et al., 2015; Zengerle et al., 2015; DeMars et al., 2018; DeMars et al., 2019). Such BET targeted degradation strategy has exhibited remarkable selectivity and efficacy. For instance, a proteomic analysis revealed that dBET1 induced an 8–10-fold decrease in BET proteins and significant downregulation in many other BET transcription-dependent proteins in treated cells (Winter et al., 2015).

**Genetic approach.** Genetic engineering animal models help identify *in vivo* protein function and its contribution to various diseases. Considering the fundamental role of BET proteins in controlling cell growth and proliferation, it is not surprising that the efforts for creating BET gene knockout strains have not succeeded. Indeed, the early genetic studies revealed that global loss of either BRD4 or BRD2 is lethal in mice (Houzelstein et al., 2002; Shang et al., 2009). BRD4 heterozygotes exhibit pre- and post-natal growth defects and various anatomical abnormalities, suggesting the BRD4’s role in fundamental cellular processes. While the BRD2 heterozygous F1 progeny were overtly normal, the subsequent generation was not. BRDT plays an essential role in regulating male germ cell differentiation. The deletion of BRDT BD1 results in male sterility, indicating the functional importance of this testis-specific gene and target potential for male contraception (Shang et al., 2007; Gaucher et al., 2012). In the past 5 years, several conditional BRD4 knockout mouse models have been generated and applied to functional analysis in various disease settings (Bolden et al., 2014; Bao et al., 2017; Lee et al., 2017; Gegonne et al., 2018; Penas et al., 2019; Duan et al., 2020).

## BET Proteins as Regulators of the Inflammatory Response

BET BDs control the assembly of histone acetylation-dependent chromatin complexes that regulate the expression of multiple inflammatory genes, suggesting that BET function is essentially required for coordinating the inflammation responses (Hargreaves et al., 2009; Cochran et al., 2019). Indeed, significant progress has been achieved in the past decade, establishing the role of BET proteins in mediating inflammation with chemical inhibitors or using genetic approaches. Early studies of BET inhibition revealed good anti-inflammatory activity, which suggests that BRD2, BRD3, and BRD4 play an essential role in orchestrating the inflammatory process (Hargreaves et al., 2009; Nicodeme et al., 2010; Belkina et al., 2013; Shi and Vakoc, 2014; Xu and Vakoc, 2014; Bao et al., 2017). In a landmark study by Nicodeme and colleagues, BET inhibition with I-BET potently suppressed the production of pro-inflammatory proteins in activated macrophages *in vivo* and protected against lipopolysaccharide (LPS)-induced lethal shock (Nicodeme et al., 2010). Treating macrophages with I-BET prevented the transcription of a specific subset of LPS-inducible genes that encode various inflammatory mediators. The absence of LPS stimulation led to minimal changes in global gene expression, suggesting selective inhibition of inflammatory gene expression by BET inhibition in this cell type. I-BET downregulates the expression of several pro-inflammatory cytokines (GM-CSF and IL-17) and upregulates the expression of several anti-inflammatory products (IL-10, LAG3, and EGR2), causing long-lasting suppression of the pro-inflammatory functions of Th1 cells (Bandukwala et al., 2012). Such suppressive effects of I-BET-762 on T-cell mediated inflammation *in vivo* were also accompanied by the reduced recruitment of macrophages. *In vitro* studies showed that genetic disruption of BRD2 or BET inhibition by JQ1 impaired mouse macrophage inflammatory responses, indicating that BRD2 is essentially required for pro-inflammatory cytokine production in macrophages (Belkina et al., 2013; Jung et al., 2015). BET protein deletion by PROTAC dBET1 robustly reduced inflammatory responses in LPS-activated microglia and aged mice subjected to ischemic stroke (DeMars et al., 2018; DeMars et al., 2019). A more recent genetic study further advanced the understanding of BET in inflammatory regulation. Mice lacking BRD4 in myeloid-lineage cells showed resistance to LPS-induced sepsis. The deletion of BRD4 reduced both the expression of LPS-induced inflammatory genes and cytokine production while enhancing the expression of a small number of LPS-suppressed genes in bone marrow-derived macrophages (Bao et al., 2017). Therefore, the critical role of BET in direct and indirect inflammatory regulation has been established using various pharmacological and genetic approaches.

The inflammatory response, a reaction of the microcirculation to injury and/or infection, is a complex but coordinated process involving multiple levels of molecules (including complement, chemokines, cytokines, free radicals, adhesion molecules, vasoactive amines, and eicosanoids), cells (including tissue macrophages, dendritic cells, lymphocytes, endothelial cells, fibroblasts, and mast cells) and physiological alterations (Fullerton and Gilroy, 2016). As indicated below, published data have recently emphasized the significant role of BET proteins in inflammation through at least two independent regulatory mechanisms: NF-κB and modulation of redox metabolism through Nrf2.

**BET and NF-κB signaling.** The nuclear factor-κB (NF-κB) transcription factor family plays a critical role in inflammation and cancer, consisting of five different DNA-binding proteins that form a variety of homo- or heterodimers: p50 (also known as NF-κB1), p52 (also known as NF-κB2), p65 (also known as RelA), cRel and RelB (Ghosh and Hayden, 2008; Fullerton and Gilroy, 2016; Campbell et al., 2021). NF-κB dimers bind to NF-κB sites at promoters or enhancers of target genes, and their transcription is regulated through the recruitment of co-activators and co-repressors (Ghosh and Hayden, 2008). Inflammatory stimuli activate signal transduction pathways (e.g., upon binding of LPS to toll-like receptor TLR4) that trigger NF-kB’s nuclear translocation, leading to the activation of inflammatory gene transcription (Ghosh and Hayden, 2008). NF-κB-mediated gene expression regulates the production of inflammatory mediators, cell proliferation and survival, development and differentiation of T cells, and maturation of dendritic cells (Taniguchi and Karin, 2018). Not surprisingly, dysregulation of NF-κB signaling significantly contributes to many inflammation-associated diseases. Consequently, the interaction between BET proteins and the NF-κB pathway has been a focus of intensive research exploring BET molecular mechanisms and pharmacological targeting.

Recent work has revealed that BET inhibitors potently suppress the LPS-induced inflammatory response, which may be related to a direct interaction between BRD4 and NF-κB (Huang et al., 2009; Nicodeme et al., 2010; Brown et al., 2014; Hah et al., 2015; Chen et al., 2016). BET inhibition by I-BET in macrophage cells significantly suppresses LPS-induced inflammatory gene transcription (Huang et al., 2009; Nicodeme et al., 2010). BRD4 increases transcriptional activation of NF-κB and the expression of a subset of NF-κB-regulated inflammatory genes in a process dependent on the binding to acetylated lysine-310 on p65. In contrast, the direct interaction of BRD4 with acetylated NF-κB subunit p65 through twin acetyl-lysine recognizing BDs of BRD4 is required for NF-κB transactivation. In addition to the direct interactions between BRD4 and NF-κB, BRD4 directly recruits cyclin-dependent kinase-9 (CDK9) to phosphorylate RNA polymerase II and facilitate the transcription of NF-κB target genes (Huang et al., 2009). Another *in vivo* study showed that NF-κB formed super-enhancers to promote rapid pro-inflammatory gene expression in a BET BD-dependent manner (Brown et al., 2014). JQ1 was shown to inhibit H. *pylori-*induced interaction between BRD4 and p65 and the recruitment of BRD4 and RNA polymerase II to the promoter and enhancer regions of inflammatory genes (Chen et al., 2016). Another report utilizing a rat spinal cord injury model, JQ1 suppressed NF-ΚB signaling activation and reduced the expression level of pro-inflammatory cytokines in microglia (Wang et al., 2019). Thus, BRD4 has been considered as a critical transcriptional regulator of NF-κB–dependent inflammatory gene expression.

A recent *in vivo* genetic study showed that, by modulating the translation of IκBα *via* the Mnk2-eIF4E pathway, BRD4 critically participates in the control of NF-κB–dependent inflammatory gene expression (Bao et al., 2017). A key observation is that BRD4 absence in myeloid-lineage cells led to enhanced expression of MAP kinase-interacting serine/threonine-protein kinase 2 (Mknk2) and activation of eukaryotic translation initiation factor 4E (eIF4E) after LPS stimulation, enhancing translation of IκBα, the negative regulator of NF-κB. The newly synthesized IκBα enters the nucleus and inhibits the binding of NF-κB to the promoter region of inflammatory genes, eventually leading to reduced inflammatory gene expression. Interestingly, the BET inhibitor, I-BET, prevents the binding of CREB-binding protein (CBP) to the promoter of IL-6 and consequently selectively regulates IL-6 production without affecting p65, suggesting the possible direct regulatory role of BET inhibition on inflammatory gene expression (Barrett et al., 2014).

**BET and Nrf2 Signaling.** The transcription factor Nrf2 is a master regulator of redox balance, inflammation, cell stress response, metabolism, and protein homeostasis (Cuadrado et al., 2019). In response to stress, the Nrf2 protein is released from Keap1-mediated repression. The accumulated Nrf2 translocates into the nucleus and binds to the antioxidant response element (ARE), activating multiple cytoprotective genes (Ma, 2013; Kumar et al., 2014; Suzuki and Yamamoto, 2015; Taguchi and Yamamoto, 2017; Bellezza et al., 2018; Yamamoto et al., 2018). To date, more than 250 Nrf2 target genes have been identified that contain the ARE in their promoter regulatory regions (Ma, 2013; Cuadrado et al., 2018; Yamamoto et al., 2018). Pathological levels of reactive oxygen species (ROS) accumulation that may impair redox signaling are closely associated with inflammation. ROS act as crucial signaling molecules during inflammation and likewise induce direct injury to the inflamed tissue. Mitochondrial dysfunction and overactivation of NADPH oxidase cause enhanced ROS production in inflammatory cells, thus activating the inflammasome and ultimately resulting in tissue and organ damage (Lopez-Armada et al., 2013).

Interestingly, both BET and Nrf2 play critical roles in inflammation and therefore are considered promising drug targets. However, the understanding of the regulatory interplay between BET and Nrf2 signaling remains incomplete. As epigenetic readers, BET proteins interact with acetylated lysine residues on histone or non-histone proteins that recognize multiple transcriptional regulators, eventually activating or repressing gene transcription (Cochran et al., 2019), suggesting the possible regulatory role of BET on Nrf2 signaling (Chatterjee and Bohmann, 2018).

Multiple studies utilizing *in vitro* oxidative stress models investigated the effects of BET knockdown by siRNA or JQ1 on oxidative damage and the induction of Nrf2 signaling and its target antioxidant genes. In response to H_2_O_2_ stimulus, BRD4 knockdown or BRD4 inhibition by JQ1 suppressed oxidative damage in cultured rat chondrocytes, as assessed by the reduction in ROS production, malondialdehyde content, and the increase in the activity of the antioxidant proteins superoxide dismutase (SOD), catalase, and glutathione peroxidase. These protective effects were accompanied by increased protein levels of Nrf2 and its target heme oxygenase-1 (HO1) (An et al., 2018). Following H_2_O_2_ exposure, BRD4 knockdown or JQ1 decreased ROS production in human embryonic kidney (HEK) 293T cells (Hussong et al., 2014), trophoblast cells (Wu et al., 2021), and primary neurons (Zhang and Xu, 2020), as well as enhanced Nrf2 activation (Zhang and Xu, 2020; Wu et al., 2021) and HO1 expression (Hussong et al., 2014). In TGFβ-stimulated corneal myofibroblast, JQ1 attenuated ROS accumulation, accompanied by increased Nrf2 nuclear translocation and its target antioxidant genes NQO1 and SOD2 expression (Qu et al., 2018). Similarly, in TGF-β-stimulated primary human pulmonary fibroblasts, JQ1 attenuated ROS production and rectified the balance between the prooxidant gene NADPH oxidase (NOX4) and the antioxidant gene SOD2, and increased Nrf2 activity (Stock et al., 2019). In podocytes exposed to high-glucose, BRD4 knockdown or JQ1 repressed ROS production and markedly activated Nrf2 signaling with associated suppression of Keap1 (Zuo et al., 2019). In cultured THP-1 human monocytic cells, knockdown of BRD2 and BRD4 or JQ1 treatment upregulated the expression of the Nrf2 target antioxidant genes HO1, NQO1, and glutamate-cysteine ligase catalytic subunit (Michaeloudes et al., 2014). Importantly, this BET inhibition-mediated protection can be abolished, at least partially, by Nrf2 knockdown (An et al., 2018; Zuo et al., 2019). Consistent with the findings above, two recent *in vivo* studies also support the regulatory role of BET proteins in Nrf2 signaling (Liang et al., 2018; Zhang and Xu, 2020). JQ1 treatment significantly improved cognitive performance and increased the expression levels of hippocampal Nrf2 and HO1 in STZ-induced diabetic rats, accompanied by decreased oxidative stress and neuroinflammation (Liang et al., 2018). *In vivo* BRD4 knockdown by siRNA ameliorated oxidative damage and inhibited macrophage infiltration into the sciatic nerve after vincristine exposure (Zhang and Xu, 2020). The inhibitory role of BET proteins on Nrf2 signaling was also reported in Drosophila (Chatterjee et al., 2016). BET proteins are recruited to ARE elements of gene promoters for NQO1 (Michaeloudes et al., 2014) and HO1 (Hussong et al., 2014; Michaeloudes et al., 2014) and bind to the *NOX4* promoter (Stock et al., 2019). JQ1 was shown to dramatically suppress the transcription of Keap1 and thereby increasing the expression of Nrf2 target antioxidant genes in primary acute myeloid leukemia cells (Huang et al., 2018). miRNA-146b-5p (miR-146b-5p) was shown to protect against oxygen/glucose deprivation-induced injury of oligodendrocyte precursor cells through regulatory mechanisms of Nrf2, while BRD4 seems to participate in this Nrf2-dependent protection (Li et al., 2019). In summary, several lines of evidence have revealed the protective role of BET inhibition against oxidative damage through upregulating the Nrf2 pathway, suggesting that BET protein inhibits Nrf2 signaling. As such, BET proteins have been identified as Nrf2 signaling repressors, which involves Keap1-dependent regulation (Huang et al., 2018; Zuo et al., 2019) and Keap1-independent regulation (Hussong et al., 2014; Michaeloudes et al., 2014; Chatterjee et al., 2016; Li et al., 2019; Stock et al., 2019). Such discoveries contribute to understanding the complex biology of BET proteins and their crosstalk with the Nrf2 pathway.

## Contribution of BET Proteins to CNS Disorders

Neuroinflammation is a significant player in the pathogenesis and progression of multiple neurological conditions, and there is a growing interest in understanding the role of BET proteins in modulating the onset and evolution of CNS inflammation (Sofroniew, 2015; Sweeney et al., 2019; Singh and Sartor, 2020; Mishra et al., 2021; Psenicka et al., 2021).

**Stroke.** Recent studies utilizing permanent and transient cerebral ischemia models have revealed the functional benefit of BET inhibition on infarct volume and neurobehavioral deficits after ischemia through inflammatory regulation and NF-κB pathway (Liu et al., 2017; DeMars et al., 2019; Zhou et al., 2019). In a permanent cerebral ischemia study using aged mice (18–20 months old), BET blockade by dBET1, a PROTAC, significantly reduces infarct volume at 48 h after stroke. dBET1 treatment improves neurological function at 24 and 48 h after the ischemic insult, as assessed using the open field test, adhesive removal test, and nesting behavior (DeMars et al., 2019). Of note, such beneficial effect of BET disruption on stroke outcomes is associated with reduced levels of brain pro-inflammatory mediators including TNF-α, CXCL1, CXCL10, CCL2, and matrix metalloproteinase-9. At 24 h after transient cerebral ischemia (2 h ischemia/reperfusion), JQ1-treated rats exhibit a marked reduction in neurological deficits, infarct volume, and expression of pro-inflammatory mediators IL-1β, IL-6, IL-17, and TNF-α in the ischemic brain (Liu et al., 2017). JQ1 remarkably attenuates nuclear NF-κB p65 levels and upregulates cytosolic IκB, indicating the suppression of NF-κB signaling by BET inhibition during ischemic injury. After transient cerebral ischemia injury (1 h ischemia and 3 days of reperfusion), JQ1 significantly reduces infarct volume, neurological deficit score, and glial activation in ischemic mice, along with significantly decreased levels of pro-inflammatory factors IL-1β, IL-6, IL-18, and TNF-α (Zhou et al., 2019).

**Spinal cord injury.** The pathophysiology of spinal cord injury (SCI) involves irreversible primary damage and reversible secondary injury. As a significant contributor to damage, inflammation has been considered a promising target to ameliorate secondary injury. BRD2, BRD3, and BRD4 mRNA are expressed in the uninjured/injured mouse spinal cord (Rudman et al., 2018). JQ1 dramatically decreases pro-inflammatory cytokine expression (such as TNF-α, IL-1β, IL-6, CCL5, and CCL2) and leukocyte recruitment to the injury site 3 days after injury. However, this benefit does not lead to improvements in locomotor activity or lesion size (Rudman et al., 2018). In a rat SCI model, BRD4 expression correlates with levels of pro-inflammatory cytokines (Wang et al., 2019). BRD4 inhibition by JQ1 represses the levels of pro-inflammatory cytokines in microglia both *in vitro* and *in vivo*, eventually leading to improvement in functional recovery, structural integrity, and neuronal loss (Wang et al., 2019). JQ1 reduces pro-inflammatory and enhances anti-inflammatory cytokine production in an SCI model in mice (Sanchez-Ventura et al., 2019). At 4 h post-lesion, JQ1 significantly reduces the expression of the pro-inflammatory cytokines IL-1β, IL-6, and TNF-α. Strikingly, IL-1β remains downregulated for over 72 h. Overall, JQ1 treatment decreases chemokine levels, whereas significant differences are observed for CCL2 at 72 h compared to vehicle controls (Sanchez-Ventura et al., 2019). Moreover, prolonged therapy with JQ1 promotes functional recovery for over 28 days (Sanchez-Ventura et al., 2019; Li et al., 2020), reduces neuropathic pain, and decreases microglia/macrophages reactivity (Sanchez-Ventura et al., 2019).

**Multiple sclerosis(MS)**. MS is an autoimmune and neurodegenerative disorder characterized by chronic inflammation, demyelination, and loss of axons and neurons (Chu et al., 2018). Using an experimental autoimmune encephalomyelitis (EAE) mouse model of MS, limited treatment with the BET inhibitor I-BET-762 inhibits the ability of T helper type 1-differentiated 2D2 T cells to induce neuroinflammation *in vivo* during the early priming phase (Bandukwala et al., 2012). Treatment with the BET inhibitor I-BET151 decreases the early clinical symptoms, which depends on cytokine production in the EAE mouse model (Barrett et al., 2014). BET BD2-selective inhibitor RVX-297 maintains anti-inflammatory properties and has therapeutic effects in preclinical EAE models of acute inflammation and autoimmunity (Jahagirdar et al., 2017).

**Seizures/Epilepsy.** It has been suggested that BRD2 gene variation confers an increased risk of juvenile myoclonic epilepsy, a common form of generalized epilepsy that starts in adolescence (Pal et al., 2003). A developmental decrease in parvalbumin-positive neurons precedes the onset of increased flurothyl-induced seizure susceptibility in the Brd2^+/−^ mouse model of juvenile myoclonic epilepsy and likely contributes to the clinical manifestations of this syndrome (McCarthy et al., 2020).

**Alzheimer’s Disease (AD).** AD is a debilitating neurodegenerative disease characterized by amyloid plaques, neurofibrillary tangles, and the presence of chronic neuroinflammation (Magistri et al., 2016). Recent reports suggest the great potential of BET inhibition for AD and other disorders associated with neuroinflammation. JQ1 treatment reduces the expression of several pro-inflammatory mediators, including IL-1β, IL-6, TNF-α, CCL2, NOS2, PTGS2, and tau phosphorylation at Ser396 in the hippocampus and frontal cortex of the 3xTg model of Alzheimer’s Disease (Magistri et al., 2016). However, this benefit does not lead to ameliorating learning and memory deficits in 7-month-old 3xTg mice (Magistri et al., 2016). In either wild-type animals or the APP/PS1 mouse AD model, JQ1 enhances cognitive performance and long-term potentiation (Benito et al., 2017). Further investigation revealed that JQ1 elicits a hippocampal gene expression profile related to ion channel activity and transcription and DNA repair (Benito et al., 2017).

## Conclusions and Future Perspectives

By using genetic and pharmacological approaches, including selective BETis and PROTACs, it is becoming clear that BET proteins play a fundamental role in regulating complex inflammatory pathways and are functionally linked to multiple CNS diseases associated with inflammation. However, our understanding of BET regulation and activity and its underlying mechanism(s) is limited. Most BETis are non-selective BD inhibitors, which hampers our ability to pinpoint the specific role of each BET protein in disease states. Studies with BET PROTACs and *in vivo* genetic methods to selectively target BET proteins in different cell types are now at a very early stage of development. Significant computational and experimental studies are needed to obtain a more dynamic global view of BET-mediated gene regulation. Of note, studies comparing how BET-dependent regulation changes between physiological and pathological conditions might confer new insights into disease mechanisms and the potential identification of novel targets for future therapeutic development.
